# Shrinking Working-Age Population and Food Demand: Evidence from Rural China

**DOI:** 10.3390/ijerph192114578

**Published:** 2022-11-07

**Authors:** Xinru Han, Ping Xue, Wenbo Zhu, Xiudong Wang, Guojing Li

**Affiliations:** 1 Institute of Agricultural Economics and Development, Chinese Academy of Agricultural Sciences, Beijing 100081, China; 2 Rural Development Institute, Chinese Academy of Social Sciences, Beijing 100732, China; 3 Institute of Agricultural Resources and Regional Planning, Chinese Academy of Agricultural Sciences, Beijing 100081, China

**Keywords:** food consumption, working-age population, heterogeneity, rural residents, China

## Abstract

China is facing a rapidly aging population, and the proportion of the working-age population (WAP) is showing a decreasing trend. In this study, we use a two-stage budgeting quadratic almost-ideal demand system framework to estimate the distribution of food demand elasticity under different proportions of the WAP in rural China. The results show that the income elasticities of rural residents’ demand for fruits and vegetables, animal products, oils and fats, and grains were 0.73, 0.65, 0.55, and 0.48, respectively. Additionally, the income elasticity of rural residents tended to increase as the household proportion of the WAP decreased. These results can provide a deeper understanding of the food consumption patterns of rural residents in China, and could be used in general or partial equilibrium models to forecast food supply and demand.

## 1. Introduction

Ending hunger in rural areas is key to achieving the second Sustainable Development Goal (SDG 2) [1,2]. The rapid evolution of the Chinese food consumption structure has accompanied economic changes in rural areas over the last four decades, e.g., cereals have been replaced with fats and animal products [3,4,5,6,7,8,9]. From 1985 to 2019, Chinese rural residents decreased their per capita consumption of grain from 257 kg to 155 kg, while their per capita consumption of meat increased from 11 kg to 25 kg.

During the same period, China has witnessed a rapid aging process [10,11,12]. In recent years, China’s working-age population (WAP; aged 15–64) has been declining in proportion to its total population. According to the medium variant of *the 2019 Revision of World Population Prospects* presented by the Population Division of the United Nations (https://population.un.org/wpp, accessed on 19 December 2020), China’s WAP proportion fell from 74.5% in 2010 to 71.2% in 2018, and is projected to decline further to 64.6% in 2035 (equivalent to the level in Japan in 2010) and to 59.8% in 2050 (equivalent to the level in Japan in 2020). Moreover, due to the variety of employment opportunities in cities, young workers continue to migrate from rural to urban areas [13], thereby resulting in lower WAP proportions in rural areas. In 2019, the rural WAP proportion was 66%, while the urban WAP proportion was 70%.

One of the effects of this demographic transition is consumption, such as that of food and medical services [14,15,16,17,18]. On the one hand, the life-cycle model of consumer behavior shows that consumption varies with age [19,20]. On the other hand, dietary recommendations for older adults differ from those for others ages due to the functional decline and health conditions of older adults [21,22,23]. Therefore, this study investigates the relationship between the demographic transition represented by the aging of family structures and food consumption, which could have significant practical implications for meeting the national food security strategy [9,24].

The estimation of food demand elasticity is an important aspect of food consumption research [25]. Researchers have constructed various models to estimate the price elasticity of food demand and the income (or expenditure) elasticity of demand for food among Chinese residents based on utility maximization and budget constraints [26,27,28,29,30,31,32]. It has been demonstrated that most income elasticity with respect to food demand in China is positive, as reviewed by Chen et al. [33]. However, traditional food demand elasticity measures only the average elasticity across all samples, not the heterogeneity between different residents. In recent years, researchers have examined the heterogeneity of food demand elasticity [9,34]. Recent studies have focused mainly on the following four aspects of food consumption heterogeneity.

The first is urban-rural heterogeneity [26,35]. Han and Chen estimated the food demand elasticity of migrant workers living in urban areas and demonstrated the heterogeneity of food demand elasticity among urban residents, rural residents, and migrant workers [28]. Second, income heterogeneity can be measured by estimating differences between the elasticity of different income groups [27,32,36,37,38]. For example, Zheng and Henneberry estimated the food demand elasticity of different groups of urban residents in Jiangsu Province in 2004 [38]. Third, demographic heterogeneity can be examined in terms of population and age structures [39,40]. In particular, heterogeneity in family characteristics is measured mainly in terms of adult equivalents, population aging, and the number of children or elderly people [41,42,43]. Fourth, external environmental heterogeneity can be assessed. Recent research has focused on regional differences [27], time differences [44], and climatic differences [45,46].

In light of the considerable differences in consumption between individuals, changes in the age structures of a family can significantly affect food consumption [39,47,48]. Therefore, in light of China’s aging population, changes in age structures are critical factors that must be considered when analyzing food demand [18,43]. To investigate the impacts of age structure on food consumption, previous studies have mainly used the population equivalent model [18,39] or other single equation models [40]. However, these single equation models may not satisfy the restrictions of symmetry, additivity, and homogeneity [25].

In this study, a two-stage estimation procedure was established to empirically study the age structure heterogeneity of food demand elasticity among Chinese rural residents. Following Law et al. [49], we chose the Working–Leser Model and the quadratic almost-ideal demand system (QUAIDS) model for the first and second stages, respectively. Because the unit value can be used as a proxy for unobserved prices, and can exhibit measurement errors, we applied a procedure for quality-adjusted prices following Cox and Wohlgenant [50] and Han and Chen [28] in order to address price endogeneity.

Therefore, the main contribution of this study is the use of a demand system model that can estimate the distribution of food demand elasticity under different proportions of the WAP. This study could provide a valuable supplement to research into the food demand elasticity of Chinese residents and population transition.

## 2. Materials and Methods

### 2.1. Data

#### 2.1.1. Data Source

**Figure 1:**
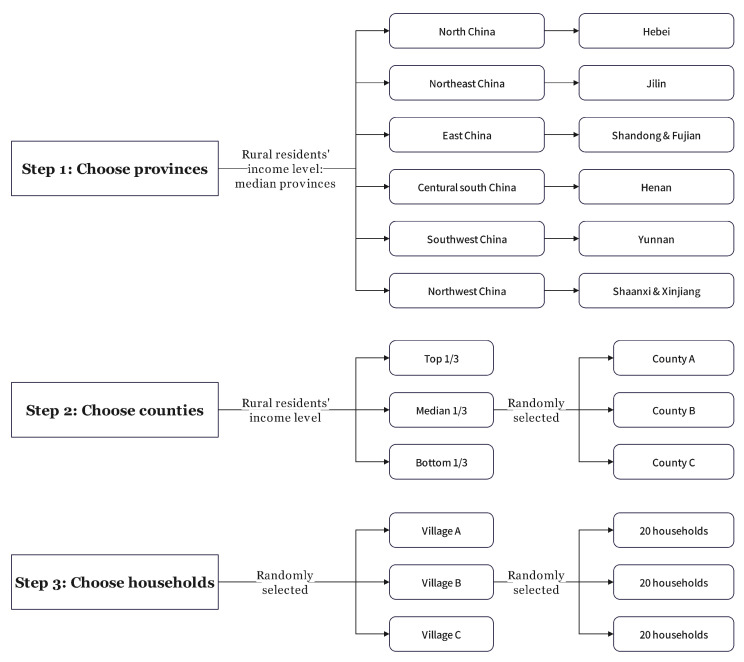
The sample selection process. Note: Nine counties were selected in Xinjiang.

In this study, we used data from the 2012–2018 Survey for Agriculture and Village Economy (SAVE) data by the Institute of Agricultural Economics and Development (IAED), Chinese Academy of Agricultural Sciences (CAAS). The dataset included 16,613 rural households from eight provinces (Hebei, Jilin, Fujian, Shandong, Henan, Yunnan, Shaanxi, and Xinjiang), 30 counties, and 295 villages (Figure 1). The data recorded the food consumption and food expenditure of the sampled households over a whole year [51,52].

In other recent studies, microdata including data from the China Health and Nutrition Survey (CHNS) [36,53] and the household survey data conducted by the National Bureau of Statistics of China (NBSC) [31,32,34] or macrodata from the NBSC have been commonly used to study the food consumption of Chinese residents [9]. CHNS data are three-day food consumption data from sampled households, and thus do not fully reflect the food consumption preference of the sampled households. Moreover, CHNS data were only published up to 2011, and may not accurately reflect the latest food consumption preferences of rural residents. While the household survey data from the NBSC cover the annual food consumption of the sampled households [54,55], they are currently not available to the public.

In the SAVE questionnaire, household food consumption is classified into staple foods, beans, and soy products; oils and fats; meat and poultry products; eggs and egg products; milk and dairy products, aquatic products; and fruits, vegetables, and fruit and vegetable products. Each category is subdivided into subcategories, such as flour and rice. To avoid having many null values, this study divided these food categories into four categories: grains (including staple foods, beans, and soy products); oils and fats; animal products (including meat and poultry products, eggs and egg products, milk and dairy products, and aquatic products), and fruits and vegetables (including fruits, vegetables, and their products). Assuming that rural households in the same region faced the same food prices [49], any missing average prices were replaced by the provincial median prices of the current year. To avoid the impact of abnormal values on the estimation results of the model, this study excluded the minimum and maximum values of the main variables, such as consumption, expenditure, and income. After data cleaning, 15,897 observations were available for use.

#### 2.1.2. Statistical Description

**Figure A1:**
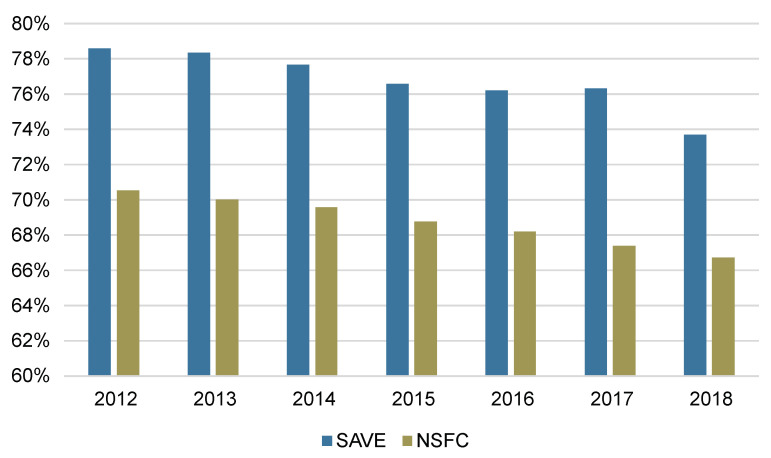
The proportion of the WAP in rural China. Source: SAVE data and the NSFC.

The proportion of household WAP was the key household characteristic variable in this study. Figure A1 shows the changes in the proportion of the WAP in rural China from 2012 to 2019. The proportion of the WAP in the sample households generally showed a downward trend, decreasing from 78.60% in 2012 to 73.68% in 2018. Although the proportion of the WAP according to the SAVE data exceeds the overall national WAP, the WAP from the SAVE data decreased as well.

The Population Division of United Nations predicts that the proportion of the WAP per household will fall to 64.6% in 2035 and 59.8% in 2050 (https://population.un.org/wpp, accessed on 19 December 2020). Thus, the WAP proportions of 60%, 65%, and 70% were chosen as the cutoff points in this study. The sample households were divided into four strata according to the proportion of the household WAP (in parentheses): G1 (0–60%), G2 (60–65%), G3 (65–70%), and G4 (70–100%). Table A1 shows that the majority of rural households had a WAP greater than 70% (10,476 households), possibly because over 50% of elderly people live alone or only with their spouses (G5) [56]. To further address the effects of an aging population on food consumption, we estimated the food demand elasticity of G5.

Table A1 shows a statistical description of the variables of each sample. First, the distribution of the per capita food expenditure of rural residents is weakly correlated with household WAP. Second, the per capita food expenditure decreased with decreasing proportion of household WAP. The average per capita food expenditure of households in the G1 group was CNY 1368.09, which was 138.39 lower than in the G4 group. The per capita total expenditure showed the same trend, i.e., it decreased with a decline in the proportion of the WAP. Therefore, the proportion of the WAP showed a positive correlation with the per capita food expenditure and the total expenditure of households.

### 2.2. Econometric Model

The basic assumption of this study was that rural residents employ a two-stage budgeting process and that their preferences are weakly separable [25]. In the first stage, rural residents allocate expenditures to food and non-food commodities; in the second stage, rural residents make decisions regarding different food items.

#### 2.2.1. Stage 1: The Working–Leser Model

In the first stage, we adopted the Working–Leser model proposed by Working and Leser [57,58]:

(1) $$w_{food}=a+b_{1}\ln m+b_{2}{\left(\ln m\right)}^{2}+u$$

where $w_{food}$ is the share of the per capita food expenditure ($m_{food}$) out of the per capita total expenditure (*m*) of the rural residents and *u* is the error term. To control the effects of household characteristic variables (Ω), such as the proportion of the WAP, on food expenditure proportions [28,29], this study defined the following:

(2) $$a=a_{0}+\Sigma_{k=1}^{K}a_{k}\Omega_{k}+u_{0}$$

#### 2.2.2. Stage 2: The QUAIDS Model

Following the initial proposal of the almost-ideal demand system (AIDS) model by Deaton and Muellbauer [25], economists have estimated the elasticity of demand for major food products in various countries and developed derivative models such as the quadratic almost-ideal demand system (QUAIDS) model [59,60]. In the second stage, we used the QUAIDS model proposed by Banks et al. [59] in the improved form proposed by Poi [61] as our model framework. Our hypothesis of the consumer expenditure function was as follows:

(3) $$e\left(p,z,u\right)=m_{0}\left(p,z,u\right)\times e^{R}\left(p,u\right)$$

where p is the price vector, z is the demographic variables vector, and *u* is the utility vector; moreover, $m_{0}\left(p,z,u\right)$ is the part of the expenditure function that reflects family features, $m_{0}\left(p,z,u\right)={\bar{m}}_{0}\left(z\right)\times \phi \left(p,z,u\right)$ (where ${\bar{m}}_{0}\left(z\right)$ measures the impact of family characteristics on expenditure), ${\bar{m}}_{0}\left(z\right)=1+\rho^{\prime }z$, ϕ(p,z,u) measures the effects of multiple factors on expenditure, $\phi \left(p,z,u\right)=\frac{\Pi_{j=1}^{k}p_{j}^{\beta_{j}}\left(\Pi_{j=1}^{k}p_{j}^{\eta_{i}^{\prime }z}-1\right)}{1/u-\Sigma_{j=1}^{k}\lambda_{j}\ln p_{j}}$, and η is an s×k matrix.

When demographic variables are not considered, the QUAIDS model can be described as follows:

(4) $$w_{i}=\alpha_{i}+\Sigma_{j=1}^{n}\gamma_{ij}\ln p_{j}+\beta_{i}\ln\left[\frac{m}{a(p)}\right]+\frac{\lambda_{i}}{b(p)}{\left\{\ln\left[\frac{m}{a(p)}\right]\right\}}^{2}+\varepsilon_{i}$$

where $w_{i}$ is the share of per capita expenditure on food *i* out of the total per capita food expenditure $W_{food}$, *n* is the number of food categories, $\varepsilon_{i}$ is the error term, $\ln a\left(p\right)=\alpha_{0}+\Sigma_{i=1}^{n}\alpha_{i}\ln p_{i}+\frac{1}{2}\Sigma_{i=1}^{n}\Sigma_{j=1}^{n}\gamma_{ij}\ln p_{i}\ln p_{j}$, $b\left(p\right)=\Pi_{i=1}^{n}p_{i}^{\beta_{i}}$; $\lambda \left(p\right)=\Sigma_{i=1}^{n}\lambda_{i}\ln p_{i}$, and $\Sigma_{i=1}^{n}\lambda_{i}=0$.

Considering demographic variables, the QUAIDS model can be modified based on Equation (4) as follows:

(5) $$w_{i}=\alpha_{i}+\Sigma_{j=1}^{n}\gamma_{ij}\ln p_{j}+\left(\beta_{i}+\eta^{\prime }z\right)\ln\left[\frac{m}{{\bar{m}}_{0}\left(z\right)}a\left(p\right)\right]+\frac{\lambda_{i}}{b(p)c(p,z)}{\left\{\ln\left[\frac{m}{{\bar{m}}_{0}}\left(z\right)a\left(p\right)\right]\right\}}^{2}+\varepsilon_{i}$$

where $c\left(p,z\right)=\Pi_{j=1}^{k}p_{j}^{\eta^{\prime }z}$, $\Sigma_{i=1}^{n}\alpha_{i}=1$, $\Sigma_{i=1}^{n}\beta_{i}=0$, $\Sigma_{i=1}^{n}\lambda_{i}=0$, $\Sigma_{j=1}^{n}\gamma_{ij}=0$, $\Sigma_{j=1}^{k}\eta_{rj}=0$ (r=1,2,⋯,s), and $\gamma_{ij}=\gamma_{ji}$(∀i≠j). If $\lambda_{i}=0$, then Equations (4) and (5) are AIDS models. We conducted a likelihood ratio test to select between the AIDS model and the QUAIDS model. Following Zheng et al. [9], we used the seemingly unrelated regression (SUR) method to estimate the QUAIDS model for n−1 food categories.

#### 2.2.3. Price Endogeneity

Because food prices were not collected by the SAVE questionnaire, we used the unit value (obtained by dividing expenditure by quantity) as a proxy for the price. Thus, there was a measurement error, and a price endogeneity problem occurred. Following Cox and Wohlgenant [50], we first regressed a unit value (uv) function model: $uv_{i}=\alpha_{i}+\Sigma_{k}\beta_{ki}X_{k}+\mu_{i}$. Then, the quality-adjusted prices (aqp) were obtained by $aqp_{i}=\alpha_{i}+{\hat{mu}}_{i}$ and used in Equation (5).

#### 2.2.4. Demand Elasticity

Considering $g_{i}=\beta_{i}+\eta^{\prime }z+\frac{2\lambda_{i}}{b(p)c(p,z)}\ln\left[\frac{m}{{\bar{m}}_{0}\left(z\right)a\left(p\right)}\right]$ and $h_{i}=\frac{\left(\beta_{i}+\eta^{\prime }z\right)\lambda_{i}}{b(p)c(p,z)}{\left\{\ln\left[\frac{m}{{\bar{m}}_{0}\left(z\right)a\left(p\right)}\right]\right\}}^{2}$, the conditional expenditure elasticity of food *i* can be described as $e_{i}=1+\frac{g_{i}}{w_{i}}$, and the conditional uncompensated (Marshallian) price elasticity of food *i* can be described as $e_{ij}^{U}=-\delta_{ij}+\frac{1}{w_{i}}\left(\gamma_{ij}-g_{i}\left(a_{i}+\Sigma_{j=1}^{n}\right)\gamma_{ij}\ln p_{j}\right)-h_{i}$. In the above, $\delta_{ij}$ is the Kronecker function. When i=j, then $\delta_{ij}=1$; otherwise, $\delta_{ij}=0$. The conditional compensated price elasticity was described as $e_{ij}^{C}=e_{ij}^{U}+e_{i}w_{j}$.

The conditional expenditure elasticity, conditional uncompensated price elasticity, and conditional compensated price elasticity of the remaining food category were $\Sigma_{i=1}^{n}e_{ij}^{U}w_{i}=-w_{j}$, $\Sigma_{i=1}e_{i}w_{i}=1$, and $\Sigma_{j=1}^{n}e_{ij}^{U}+e_{i}=0$, respectively. According to Equations (1) and (2), the unconditional expenditure elasticity (income elasticity) is $E_{i}=\left[\frac{b_{1}+2b_{2}\ln m}{W_{food}}+1\right]e_{i}$.

## 3. Results

### 3.1. Model Estimation and Selection

Table A2 reports the estimates of the Working–Leser model in the first stage. First, the coefficient of the log of the per capita total expenditure (lnm) was significant at the 1% level and positive. Second, the coefficient of the squared term of lnm was significant at the 1% level and negative. Furthermore, the coefficient of the proportion of household WAP was significant at the 1% level, as expected.

The estimation results from the demand system models in the second stage are reported in Table A3. Columns (1) and (2) show the estimations from the standard AIDS model and standard QUAIDS model, respectively, derived from Equation (4). Columns (3)–(5) show the estimations from the QUAIDS model derived from Equation (5). In Column (3), we added the proportion of the WAP as a demographic variable.

In the second phase of our food consumption estimation, we tested the joint significance of the parameter $\lambda_{i}$ in Equation (5) to choose between the AIDS and QUAIDS models. If all $\lambda_{i}$ are 0, the AIDS model should be used; otherwise, the QUAIDS model should be used (as $\Sigma_{i=1}^{n}\lambda_{i}=0$, only three λ values need to be tested for equality to 0 at the same time). The test results showed $\chi^{2}\left(3\right)=60.28$, with a probability of 0.00, which leads us to reject the null hypothesis that all $\lambda_{i}=0$ and indicates that the QUAIDS model should be used. Therefore, the follow-up analysis of this study was based on the estimation results from the QUAIDS model.

The estimation results from the AIDS and QUAIDS models are summarized in Table A3. The estimation results showed that more than two thirds of the variables in all models were statistically significant. The estimation results from the standard AIDS model (Column 1), the standard QUAIDS model (QUAIDS1, Column 2), the household characteristics QUAIDS model (QUAIDS2, excluding the provincial and year dummy variables, Column 3), the household characteristics QUAIDS model (QUAIDS3, including the provincial and year dummy variables, Column 4) were not significantly different. These results indicate that the estimation results in this study are robust.

### 3.2. Income Elasticity and Price Elasticity

Relying on the estimation results from the Working–Leser model and the household characteristics QUAIDS model (including the provincial and year dummy variables), we then calculated the conditional expenditure elasticity, income elasticity, and conditional uncompensated own-price elasticity of the whole sample and the four WAP groups. The results are summarized in Table 1 and Table 2.

The elasticity estimation results presented in Table 1 show that the significance level of all elasticity values was 1%. Among the estimated income elasticity values of the whole sample, the income elasticity of fruits and vegetables and animal products among rural residents was relatively large (0.73 and 0.65, respectively), whereas the income elasticity of oils and fats and grains was relatively small (0.55 and 0.48, respectively). In addition, the elasticity estimation results presented in Table 2 show that the significance level of all elasticity values was 1%. As indicated by the conditional uncompensated own-price elasticity of all samples, rural residents were most sensitive to price changes for fruits and vegetables (with an absolute value of own-price elasticity of 0.95), followed by animal products and grain products (with absolute values of own-price elasticity of 0.88 and 0.82, respectively). Rural residents were the least sensitive to price changes for oil and fat products (with an absolute value of own-price elasticity of 0.5).

First, when we stratified the estimation results according to the proportion of household WAP, the flexibility of food expenditure in the first stage indicated that the elasticity of food expenditure among rural residents tended to increase as the proportion of household WAP declined. The food expenditure elasticity of the G1 group (household WAP proportion of less than 50%) was 0.03 higher than the food expenditure elasticity of the G4 group (household WAP proportion of greater than 90%). These results imply that as the proportion of household WAP in China declined, rural residents tended to spend most of their budgets on food. This result suggests that over a life cycle, the pattern of food expenditure at home is high at either end and low in the middle [42].

Second, the conditional expenditure elasticity of the four food categories among rural residents was heterogeneous in terms age structure. The expenditure elasticity for grain was 0.04 higher in the G1 group than that of the G4 group, while the expenditure elasticity of demand for oil and fat products was 0.05 lower in the G1 group than the G4 group. Among both groups, the expenditure elasticity of demand for animal products and fruit and vegetable products were almost identical. The G2 group’s expenditure elasticity of demand for oils and fats was significantly higher than that of the other groups, and the expenditure elasticity of demand of the G2 group for fruits and vegetables was significantly lower than that of other groups.

Third, the comprehensive results for food expenditure flexibility in the first stage of our calculation and the conditional expenditure elasticity in the second stage showed a trend of increasing income elasticity among rural residents as the proportion of household WAP decreased. Compared with the G4 group, the G1 group had a 0.06 higher income elasticity of grain demand, a 0.04 higher income elasticity of demand for animal products, and a 0.04 higher income elasticity of demand for fruits and vegetables.

Unlike in the case of income elasticity, the correlation between the own-price elasticity of food consumption among rural residents and the proportion of the WAP was relatively small. The own-price elasticity of grain, oils and fats, and fruits and vegetables in the G1–G4 groups was almost the same, while the own-price elasticity of oils and fats differed slightly. These results indicate that the proportion of household WAP might not be a key factor in the own-price elasticity of food consumption among rural residents. With the decline in the proportion of household WAP, the own-price elasticity of food consumption remained almost unchanged among rural residents.

### 3.3. Food Demand Elasticity among the Elderly Population

Because the food consumption characteristics of elderly people living alone or with their spouses (G5) are significantly different from those of other groups, this study analyzed the food demand characteristics of the G5 group. Due to the impact of China’s one-child policy, the population trend in China is expected to continue to worsen in the future, with the proportion of elderly people who live alone or with their spouses likewise continuing to rise among social and family groups [10,62]. Due to factors such as age and income level, there are differences in consumption between elderly and young people. According to our data, the sample size of G5 was 652, which accounted for 4.1% of all sample households. The average Engel’s coefficient of G5 was 34.2%, which was 0.6 percentage points higher than other households. This suggests that the food demands of elderly people who live alone or with their spouses are different from other households in our sample.

In addition, with the aggravation of the problems associated with aging in China, the proportion of the WAP is decreasing. As a result, the G1 group is increasing as well. Therefore, this section focuses on the relevant characteristics of the G1 population. Table 3 shows that the income elasticity of demand for oils and fats and animal products among elderly people living alone or with their spouses (0.53 and 0.67, respectively) was lower than that among the G1 population (0.55 and 0.68, respectively), and the income elasticity with respect to elderly people’s demand for fruits and vegetables (0.79) was higher than that of the G1 population (0.76). These results indicate that compared to the G1 population, the elderly population’s consumption of fruits and vegetables among the elderly population was relatively sensitive to changes in income. Therefore, with the intensification of aging, the guarantee of a supply of fruits and vegetables requires extra attention.

### 3.4. Income Elasticity of Food Demand among Elderly Populations with Different Income Levels

**Figure 2:**
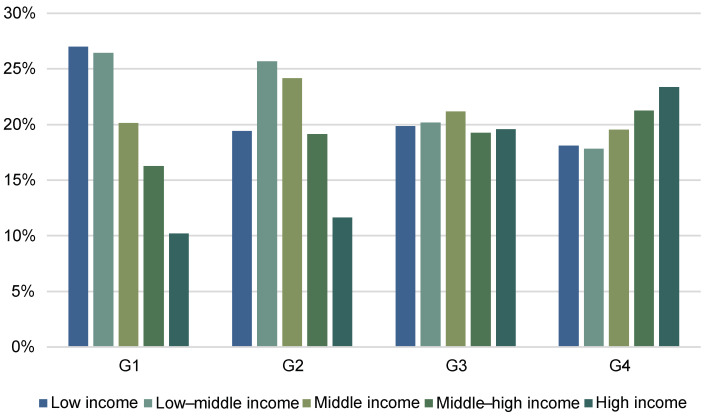
The income distribution of the four groups with different proportions of WAP. Note: Low, middle, low–middle, middle, middle–high, and high incomes represent the 0–20th, 20–40th, 40–60th, 60–80th, and 80–100th percentiles of the per capita income of the whole sample, respectively. G1 indicates a WAP proportion of 0–60%; G2 indicates a WAP proportion of WAP of 60–65%; G3 indicates a WAP proportion of 65–70%; G4 indicates a WAP proportion of 70–100%.

Recent studies have found that the income elasticity of food demand decreases as income increases [31,32,36]. In order to verify the heterogeneity of the income of populations with different proportions of WAP, the four WAP groups were divided into five equal groups according to income. The income distribution results are shown in Figure 2. There was a certain income heterogeneity between the four WAP groups, with the G1 group representing mainly low income and low–middle incomes, consistent with the life cycle hypothesis.

Furthermore, the G1 group was subdivided according to income to estimate the income elasticity of food demand. These results are summarized in Table 4. The estimation results indicate that within the G1 group, the income elasticity of food demand decreased with increasing income. For example, the income elasticity of grain demand in the low income subgroup of G1 was 0.02 higher than that in the high income subgroup. The income elasticity for food demand in the whole G1 sample was close to that of the middle and high income subgroups of G1. Therefore, there were differences in the income elasticity of food demand among elderly populations with different income levels.

## 4. Discussion

Based on the estimation results presented in Table 1 and Table 4, it can be seen, first, that as income increased, the food consumption of Chinese rural residents showed an increasing trend; additionally, rural residents tended to consume more vegetables, fruits, and animal products. This suggests that the pressure on China’s food security is likely to persist. In addition, compared to the existing estimates of income elasticity and conditional uncompensated own-price elasticity among Chinese rural residents (see Table IV in Han and Chen [28]), the estimated results in this study did not have abnormal values.

Second, in the context of accelerated population aging and high income growth rates, it is necessary to simultaneously consider the heterogeneity of family age structures and income distributions in order to accurately predict the future food demand among rural Chinese residents. Furthermore, due to the rapid advancement of urbanization and significant differences in food consumption characteristics between urban and rural residents [28], it is necessary to set up three-dimensional scenarios covering the urbanization rate, age structure, and income distribution in order to make food demand forecasts for China. Existing equilibrium models usually only include the urbanization rate [63,64,65,66]; as such, these models may not accurately reflect the level of food security within the complex domestic and international conditions of the new era.

With the start of a new journey towards building a fully modernized socialist country, China faces the challenges of rapid urbanization and an aging population. The income levels of urban and rural residents are increasing at a medium to high speed. Within this complex context, it is necessary to obtain a more comprehensive understanding of the food consumption patterns of rural residents in order to ensure food security in China. The findings of this study can provide the following insights.

First, the government needs to further strengthen the implementation of the "vegetable basket project" in rural areas to meet the demand for vegetables, fruits, and animal products among rural residents and avoid large fluctuations in the prices of "vegetable basket" products. Food consumption data from rural residents need to be further monitored and shared in order to support precise and scientific decision-making. Second, the equilibrium model for predicting food supply and demand should be adjusted according to the results of this study in order to avoid the excessive simplification of the demand equation and consequent bias in the results.

It is important to note that this study had certain limitations. First, we were unable to analyze food expenditure away from home due to a lack of data. Second, the sample coverage was narrow. Third, the sample sizes of certain subgroups were relatively small.

## 5. Conclusions and Future Directions

Relying on SAVE data from 2012 to 2018 provided by the Institute of Agricultural Economics and Development, Chinese Academy of Agricultural Sciences, we constructed a Working–Leser QUAIDS model to empirically study the heterogeneity in terms of household age structure of food demand elasticity among rural residents in China.

First, the income elasticity of demand for fruits and vegetables, animal products, oils and fats, and grains among rural residents was 0.73, 0.65, 0.55, and 0.48, respectively. Rural residents’ conditional uncompensated own-price elasticity was −0.95, −0.88, −0.50, and −0.82, respectively, indicating that demand for food among rural residents continues to increase with income growth, and that it is the most sensitive to price changes for fruits and vegetables. Second, the income elasticity of rural residents tended to increase as the proportion of household WAP decreased; households with a WAP ≤ 60% had higher income elasticity with respect to food demand than those with a WAP ≥ 70%. Third, as the proportion of household WAP declined, the own-price elasticity of food consumption among rural residents remained almost unchanged. In addition, the income distribution heterogeneity within populations with different WAP levels followed the general rule in that the income elasticity of food consumption declined with increasing income.

To the best of our knowledge, this is the first study to investigate the distribution of food demand elasticity in rural China under different proportions of WAP. In the future, research could continue to concentrate on how food demand elasticity is distributed across urban residents in relation to their age structures, how food consumption away from home differs between urban and rural residents with differing age structures, and how to predict food supply and demand according to the age structure of the population.

## Figures and Tables

**Table 1:** Estimated conditional expenditure elasticity and income elasticity.

	GR	OF	AP	FV	FEE in the 1st Stage
Conditional expenditure elasticity					
All samples	0.79 (0.00)	0.90 (0.00)	1.06 (0.00)	1.18 (0.00)	0.62 (0.00)
G1	0.82 (0.00)	0.86 (0.00)	1.05 (0.00)	1.18 (0.01)	0.65 (0.00)
G2	0.84 (0.00)	0.96 (0.00)	1.04 (0.00)	1.11 (0.00)	0.66 (0.00)
G3	0.79 (0.00)	0.89 (0.00)	1.06 (0.00)	1.18 (0.00)	0.63 (0.00)
G4	0.78 (0.00)	0.91 (0.00)	1.06 (0.00)	1.19 (0.00)	0.61 (0.00)
Income elasticity					
All samples	0.48 (0.00)	0.55 (0.00)	0.65 (0.00)	0.73 (0.00)	
G1	0.53 (0.00)	0.55 (0.00)	0.68 (0.00)	0.76 (0.00)	
G2	0.55 (0.00)	0.63 (0.00)	0.68 (0.00)	0.73 (0.00)	
G3	0.49 (0.00)	0.56 (0.00)	0.66 (0.00)	0.74 (0.00)	
G4	0.47 (0.00)	0.55 (0.00)	0.64 (0.00)	0.72 (0.00)	

Note: Standard errors are shown in parentheses; the first-stage food expenditure elasticity (FEE) of the four groups is the estimated value of food expenditure elasticity based on the proportional expenditure on the four food categories out of the average total expenditure of each group; G1 indicates aWAP proportion of 0–60%; G2 indicates a WAP pro-portion of WAP of 60–65%; G3 indicates a WAP proportion of 65–70%; G4 indi-cates a WAP proportion of 70–100%; GR = grain, OF = oils and fats, AP = animal products, and FV = fruits and vegetables.

**Table 2:** Estimated conditional uncompensated own-price elasticity.

	GR	OF	AP	FV
All samples	−0.82 (0.00)	−0.50 (0.01)	−0.88 (0.00)	−0.95 (0.01)
G1	−0.83 (0.00)	−0.49 (0.01)	−0.88 (0.00)	−0.95 (0.01)
G2	−0.83 (0.00)	−0.52 (0.00)	−0.87 (0.00)	−0.94 (0.00)
G3	−0.83 (0.00)	−0.46 (0.01)	−0.88 (0.00)	−0.95 (0.00)
G4	−0.82 (0.00)	−0.51 (0.00)	−0.88 (0.00)	−0.95 (0.01)

Note: Standard errors are shown in parentheses; G1 indicates aWAP proportion of 0–60%; G2 indicates aWAP proportion of WAP of 60–65%; G3 indicates a WAP proportion of 65–70%; G4 indicates a WAP proportion of 70–100%; GR = grain, OF = oils and fats, AP = animal products, and FV = fruits and vegetables.

**Table 3:** Estimated conditional expenditure elasticity and income elasticity among the elderly population.

	GR	OF	AP	FV	FEE in the 1st Stage
Conditional expenditure elasticity	0.82(0.01)	0.83(0.01)	1.05(0.00)	1.24(0.01)	0.64(0.00)
Income elasticity	0.53(0.01)	0.53(0.01)	0.67(0.00)	0.79(0.01)	

Note: standard errors are shown in parentheses. FEE = food expenditure elasticity, GR = grain, OF = oils and fats, AP = animal products, and FV = fruits and vegetables.

**Table 4:** Estimation results for income elasticity among the different subgroups of the G1 group.

	GR	OF	AP	FV	Obs.
Low income	0.56 (0.00)	0.57 (0.01)	0.69 (0.00)	0.79 (0.01)	794
Low–middle income	0.55 (0.00)	0.57 (0.00)	0.70 (0.00)	0.81 (0.01)	778
Middle income	0.54 (0.00)	0.59 (0.00)	0.68 (0.00)	0.74 (0.01)	592
Middle–high income	0.53 (0.00)	0.57 (0.00)	0.69 (0.00)	0.76 (0.00)	479
High income	0.47 (0.00)	0.51 (0.01)	0.67 (0.00)	0.75 (0.01)	300
Whole G1 group	0.53 (0.00)	0.55 (0.00)	0.68 (0.00)	0.76 (0.00)	2943

Note: Standard errors are shown in parentheses. GR = grain, OF = oils and fats, AP = animal products, and FV = fruits and vegetables.

## Data Availability

The data presented in this study are available upon request from the corresponding author.

## Appendix A

**Table A1:** Statistical description of major variables.

Category	Unit	WAP Strata				All
		G1 0–60%	G2 60–65%	G3 65–70%	G4 70–100%	
Per capita quantities consumed at home						
Grain (GR)	Kg	78.40 (61.12)	75.08 (62.47)	84.59 (61.42)	87.18 (64.07)	84.76 (63.28)
Oils and fats (OF)	Kg	12.24 (8.07)	11.87 (7.70)	12.27 (8.21)	13.80 (8.84)	13.26 (8.62)
Animal products (AP)	Kg	39.48 (32.32)	35.19 (26.45)	40.56 (29.61)	43.86 (33.89)	42.31 (32.94)
Fruits and vegetables (FV)	Kg	61.66 (66.40)	59.44 (57.12)	62.85 (66.21)	63.08 (66.99)	62.64 (66.41)
Price (unit value)						
Grain (GR)	CNY/kg	5.12 (5.03)	5.01 (4.91)	4.88 (4.35)	4.93 (4.27)	4.96 (4.46)
Oils and fats (OF)	CNY/kg	12.41 (6.90)	12.47 (5.77)	12.58 (6.76)	13.35 (8.82)	13.05 (8.17)
Animal products (AP)	CNY/kg	20.52 (15.49)	20.66 (11.52)	21.14 (14.90)	21.27 (14.57)	21.09 (14.67)
Fruits and vegetables (FV)	CNY/kg	5.00 (3.50)	4.85 (2.87)	5.34 (3.99)	5.45 (4.47)	5.33 (4.20)
Household characteristic						
Per capita food expenditure	CNY 1000	1.37 (0.72)	1.29 (0.70)	1.47 (0.77)	1.57 (0.79)	1.51 (0.78)
Per capita total expenditure	CNY 1000	4.31 (2.45)	4.08 (1.99)	4.71 (2.46)	5.15 (2.80)	4.90 (2.70)
Proportion of the WAP		0.36 (0.21)	0.60 (0.01)	0.67 (0.00)	0.93 (0.11)	0.78 (0.26)
Observations		2943	654	1824	10,476	15,897

Note: The income and price variables were all deflated by the national consumer price index (2012 = 100) data from the NBSC; USD 1 = CNY 6.62 in 2018; standard deviations are shown in parentheses; G1 indicates aWAP proportion of 0–60%; G2 indicates a WAP proportion of WAP of 60–65%; G3 indicates a WAP proportion of 65–70%; G4 indicates a WAP proportion of 70–100%.

**Table A2:** Estimation results from the Working–Leser model.

	Estimated Result	Bootstrap Standard Error
lnm	0.32 ***	(0.05)
${\left(\ln m\right)}^{2}$	−0.03 ***	(0.00)
Proportion of household WAP	0.02 ***	(0.00)
Provincial dummy variables	Y	Y
Year dummy variables	Y	Y
Number of samples	15,897	15,897

Notes: *** *p* < 0.01.

**Table A3:** Estimation results from the demand system model in the second stage.

Parameters	AIDS	QUAIDS1	QUAIDS2	QUAID3
	(1)	(2)	(3)	(4)
$\alpha_{1}$	0.28 ***	0.28 ***	0.28 ***	0.29 ***
$\alpha_{2}$	0.10 ***	0.10 ***	0.10 ***	0.11 ***
$\alpha_{3}$	0.42 ***	0.42 ***	0.42 ***	0.40 ***
$\alpha_{4}$	0.19 ***	0.19 ***	0.20 ***	0.20 ***
$\beta_{1}$	−0.02 ***	−0.02 ***	−0.01	−0.03 ***
$\beta_{2}$	−0.04 ***	−0.04 ***	−0.04 ***	−0.04 ***
$\beta_{3}$	0.01 ***	0.01 ***	0.00	0.02 ***
$\beta_{4}$	0.05 ***	0.05 ***	0.05 ***	0.04 ***
$\gamma_{11}$	0.02 ***	0.03 ***	0.03 ***	0.03 ***
$\gamma_{21}$	−0.02 ***	−0.02 ***	−0.02 ***	−0.01 ***
$\gamma_{31}$	−0.02 ***	−0.02 ***	−0.02 ***	−0.02 ***
$\gamma_{41}$	0.01 ***	0.01 ***	0.01 ***	0.01 ***
$\gamma_{22}$	0.06 ***	0.06 ***	0.06 ***	0.06 ***
$\gamma_{32}$	−0.04 ***	−0.04 ***	−0.04 ***	−0.04 ***
$\gamma_{42}$	−0.01 ***	−0.01 ***	−0.01 ***	−0.01 ***
$\gamma_{33}$	0.07 ***	0.07 ***	0.07 ***	0.07 ***
$\gamma_{43}$	−0.01 ***	−0.01 ***	−0.01 ***	−0.01 ***
$\gamma_{44}$	0.01 ***	0.01 ***	0.01 ***	0.01 ***
$\eta_{1}$			−0.02 *	−0.01 ***
$\eta_{2}$			0.00	0.00
$\eta_{3}$			0.01	0.01 ***
$\eta_{4}$			0.01	0.00 ***
ρ			0.32 ***	0.00
$\lambda_{1}$		−0.01 *	−0.01 *	0.00 ***
$\lambda_{2}$		0.00	0.00	−0.01 ***
$\lambda_{3}$		0.00	0.00	−0.00 ***
$\lambda_{4}$		0.00 **	0.01 **	0.01 ***
Provincial dummy variable	N	N	N	Y
Year dummy variable	N	N	N	Y
Number of samples	15,897	15,897	15,897	15,897

Notes: ***, **, and * indicate significance at the 1%, 5%, and 10% levels, respectively.
